# Cyclodextrin/PVP-Based Nanofibers with *Rhadiola rosea* Extract as a New System for Increasing Bioavailability of Active Components

**DOI:** 10.3390/molecules30163359

**Published:** 2025-08-13

**Authors:** Maciej Jaskólski, Magdalena Paczkowska-Walendowska, Zuzanna Rybarczyk, Natalia Rosiak, Andrzej Miklaszewski, Judyta Cielecka-Piontek

**Affiliations:** 1 Department of Pharmacognosy and Biomaterials, Poznan University of Medical Sciences, Rokietnicka 3, 60-806 Poznan, Poland; jaskolski.mj@gmail.com (M.J.); rybarczyk.zuzanna@gmail.com (Z.R.); nrosiak@ump.edu.pl (N.R.); jpiontek@ump.edu.pl (J.C.-P.); 2 Faculty of Mechanical Engineering and Management, Institute of Materials Science and Engineering, Poznan University of Technology, 60-965 Poznan, Poland; andrzej.miklaszewski@put.poznan.pl

**Keywords:** *Rhodiola rosea*, electrospun nanofibers, cyclodextrins, polyvinylpyrrolidone, bioavailability, salidroside, rosarin

## Abstract

The present study aimed to optimize the extraction process for *Rhodiola rosea* root, then develop and optimize electrospun nanofiber systems containing extract to enhance the bioavailability of its active compounds, salidroside and rosarin. Using a Design of Experiments (DoE) approach, nanofibers were prepared with varying ratios of polyvinylpyrrolidone (PVP) and hydroxypropyl-cyclodextrins (HPαCD, HPβCD, HPγCD). The systems were comprehensively characterized in terms of morphology, content of active compounds, dissolution rate, permeability, mucoadhesion, antioxidant, and anti-inflammatory activities. The results showed that nanofiber formulations significantly improved the dissolution and permeability of salidroside and rosarin compared to the crude extract. The antioxidant properties were notably enhanced, while the anti-inflammatory activity varied depending on composition. The formulation containing 3 g HPβCD and 2.5 g PVP demonstrated the most favorable balance of functional and technological properties. Principal Component Analysis (PCA) and correlation matrix analysis confirmed that system composition strongly influenced the interrelationships between technological parameters and bioactivity. These findings indicate that electrospun nanofibers based on cyclodextrin-PVP matrices are a promising preclinical strategy for improving the delivery of *Rhodiola rosea* bioactives.

## 1. Introduction

*Rhodiola rosea* L. (*Crassulaceae*), commonly known as golden root, roseroot, or Arctic root, is a traditional medicinal plant that has been used for centuries in Europe and Asia as a natural adaptogen to combat fatigue, improve physical and mental performance, and support resilience to stress [1]. The therapeutic potential of *R. rosea* is primarily attributed to its rich phytochemical composition, dominated by phenolic glycosides such as salidroside, and a group of cinnamyl alcohol glycosides collectively known as rosavins (rosavin, rosarin, and rosin) [2]. Other important constituents include p-tyrosol and minor polyphenolic compounds, which together contribute to the plant’s broad spectrum of pharmacological activities [3]. These bioactives have been extensively studied for their antioxidant, anti-inflammatory, neuroprotective, cardioprotective, and anticancer properties [4]. Salidroside and rosavins have been shown to act as potent scavengers of reactive oxygen species (ROS), inhibitors of pro-inflammatory enzymes (e.g., hyaluronidase, 5-lipoxygenase), and modulators of cellular stress pathways [5,6].

Despite its promising biological activity, the clinical application of *R. rosea* extract is limited by the poor bioavailability of its key active compounds. The bioavailability of phytochemicals such as salidroside and rosavins is hindered by several factors, including relatively low aqueous solubility (around 5 mg/mL for rosavin and salidroside), poor absorption in the intestines with a low apparent permeability across cell monolayers, and chemical instability under physiological conditions [7,8]. As a result, after oral administration, only a small fraction of these compounds reaches systemic circulation in bioactive form, reducing their therapeutic efficacy. This limitation poses a significant challenge for the development of effective *R. rosea*-based health products and highlights the need for advanced delivery strategies that can overcome these barriers [9].

To address these challenges, various formulation strategies have been investigated to enhance the bioavailability of *R. rosea* actives. Conventional approaches include the use of co-solvents [10] and inclusion complexes with cyclodextrins [6,11]. Cyclodextrins have been widely studied for their ability to form inclusion complexes with hydrophobic molecules, improving their solubility, stability, and dissolution rate [12]. Polyvinylpyrrolidone (PVP), a hydrophilic polymer with excellent film-forming and solubilizing properties, has also been employed as a carrier matrix for poorly soluble natural compounds [13]. However, while these methods provide some improvement, they may not sufficiently enhance both solubility and permeability simultaneously.

Electrospinning has emerged as a novel and highly promising technique for the development of advanced drug delivery systems [14]. This versatile method enables the production of nanofibrous materials characterized by a high surface-to-volume ratio, tunable porosity, and the capacity to encapsulate bioactive compounds in an amorphous or molecularly dispersed form [15,16]. These features facilitate rapid dissolution, enhanced diffusion, and improved stability of incorporated actives. Electrospun nanofibers composed of PVP and cyclodextrins can combine the solubilization benefits of inclusion complex formation with the structural advantages of nanofibers, providing a unique platform for overcoming the bioavailability challenges of phytochemicals [17].

Although electrospinning has been successfully applied to improve the delivery of various natural compounds, including polyphenols, flavonoids, and alkaloids, comprehensive studies on its application to *Rhodiola rosea* extracts remain scarce. A recent study demonstrated the successful incorporation of salidroside into a coaxial electrospun polycaprolactone/gelatin nanofiber membrane alongside cryptotanshinone, showing synergistic effects in promoting vascularization and osteogenesis [18]. However, this research focused on a different therapeutic indication and did not address key pharmaceutical challenges related to *R. rosea* actives—such as enhancing the solubility, permeability, and overall bioactivity of compounds like salidroside and rosavins. This gap highlights the need for targeted research into the design and characterization of electrospun nanofiber systems specifically tailored to optimize the delivery of *R. rosea* constituents.

In this context, the present study aimed to optimize the extraction process of *Rhodiola rosea*, then develop electrospun nanofiber systems containing extract incorporated into PVP and cyclodextrin matrices. Using a design of experiments (DoE) approach, we systematically investigated the influence of formulation parameters on the technological characteristics (morphology, process efficiency, mucoadhesion) and functional properties (release rate, permeability, antioxidant and anti-inflammatory activity) of the nanofibers. The goal was to propose an effective delivery strategy capable of enhancing the bioavailability of *R. rosea* bioactives and unlocking their therapeutic potential for future pharmaceutical and nutraceutical applications.

## 2. Results and Discussion

### 2.1. Optimization of the Radiola Extract Extraction Process and Investigation of Its Biological Activity

**Figure 1:**
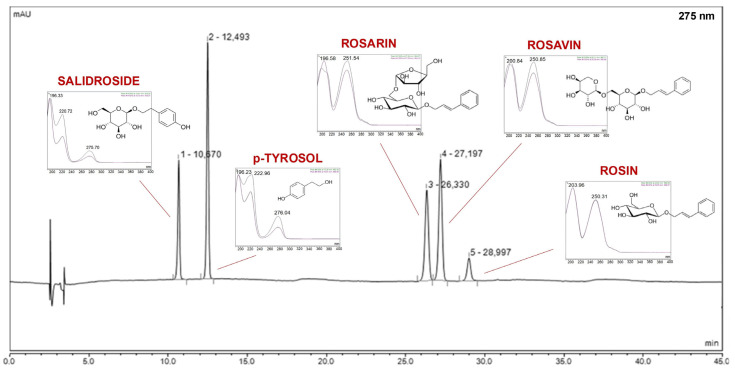
Chromatogram of substance standards.

The prepared extracts were tested for the total polyphenol content (Table 1). To identify and determine the active compounds in previously prepared extracts from the root of *Rhodiola rosea*, a high-performance liquid chromatography method was developed. This method allowed for qualitative and quantitative analysis of rosavins (rosavin, rosin, rosarin), p-tyrosol, and salidroside (Figure 1).

**Figure 2:**
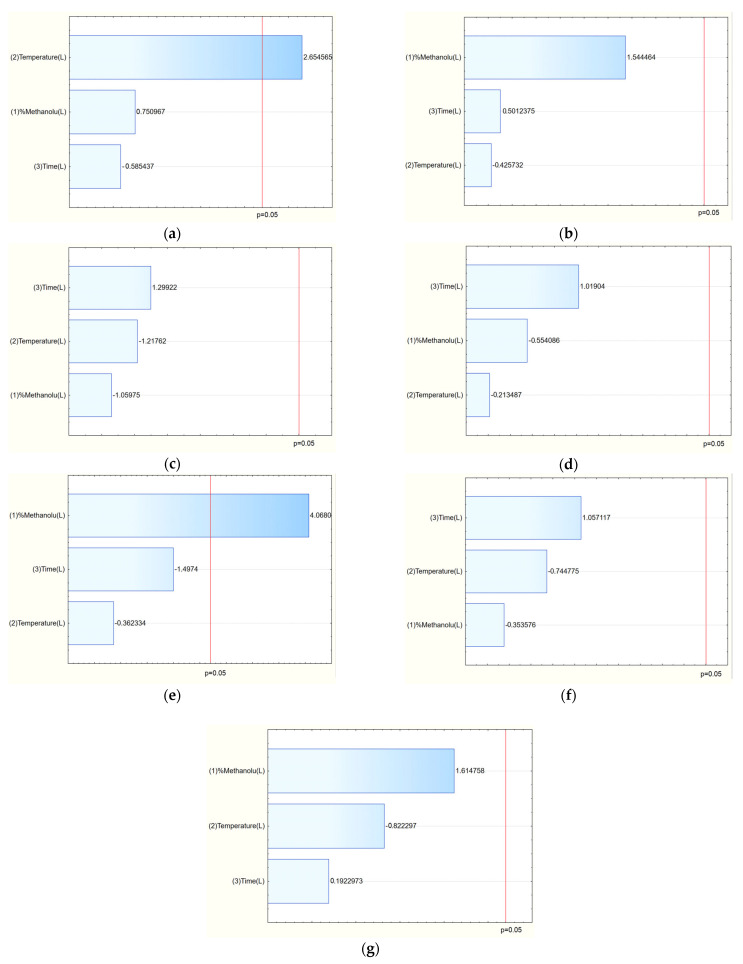
Pareto plots for TPC (**a**), salidroside content (**b**), p-tyrosol (**c**), rosavin (**d**), rosarin (**e**), rosin (**f**) and sum of active components (**g**).

The TPC values ranged from 51.69 mg GAE/g (E7) to 94.04 mg GAE/g (E14), indicating that extraction conditions significantly influenced phenolic yield. The Pareto analysis revealed that temperature had a statistically significant positive effect on TPC: higher temperatures enhanced the extraction efficiency of polyphenolic compounds (Figure 2a).

Chromatographic analysis identified and quantified salidroside, p-tyrosol, rosavin, rosarin, and rosin. Among these, the sum of active components varied across extraction conditions, with the highest total content observed in E8 (42.21 mg/g) and E14 (41.14 mg/g). The Pareto plots indicated that methanol concentration in the extraction mixture was the key factor influencing rosarin content, with higher methanol levels generally increasing its extraction (Figure 2e). Similarly, for the sum of active components, a clear trend was observed: increasing methanol concentration in the extraction solvent correlated with a higher total content of active compounds (Figure 2g).

The antioxidant activity study was performed using the DPPH radical, while anti-inflammatory activity was expressed as the degree of inhibition of hyaluronidase enzyme activity (Table 2). The extracts with higher total polyphenol content (TPC) and greater concentrations of active components, particularly rosavins, generally exhibited stronger antioxidant and anti-inflammatory effects. For example, extract E14, which recorded the highest TPC (94.04 mg GAE/g) and one of the highest sums of active compounds (41.14 mg/g), corresponded with the best antioxidant performance. Similarly, extracts E4 and E14, both with elevated TPC and active compound content, showed superior hyaluronidase inhibition.

**Figure 3:**
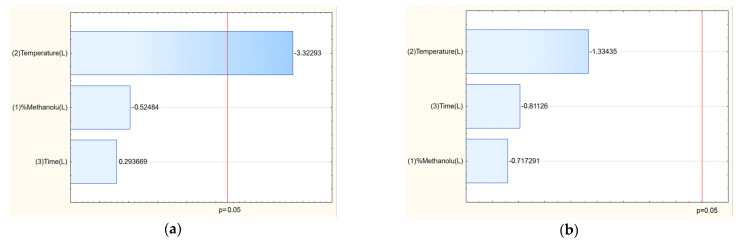
Pareto plots for antioxidant (**a**) and anti-inflammatory (**b**) activities.

Analysis of the Pareto plots demonstrated that temperature (significantly) and methanol concentration influenced the biological activity of the extracts (Figure 3). Specifically, with increasing methanol concentration and temperature during extraction, IC_50_ values for antioxidant and anti-inflammatory activities decreased, indicating enhanced bioactivity of the extracts. This trend aligns with the enhanced extraction of polyphenols and bioactive constituents under these conditions.

From the perspective of phytochemical research, these findings are consistent with existing literature. Polyphenols, rosavins, and salidroside have been widely documented to contribute to the antioxidant and anti-inflammatory potential of *Rhodiola rosea* extracts [3,19,20]. Rosavin and salidroside act as scavengers of reactive oxygen species (ROS) and as inhibitors of pro-inflammatory enzymes, including hyaluronidase [21]. Although the above study only analyzed the effect of different methanol concentrations, temperature and extraction time, the obtained results are consistent with the literature data, which showed higher efficiency of alcohol extracts compared to water extracts, which is confirmed by the higher efficiency of organic solvents in the extraction of compounds with antioxidant properties [22]. Importantly, the 5-lipoxygenase inhibitory activity of the main constituents, particularly rosiridin, kenposide A, and rosavins, was demonstrated, confirming these compounds as key contributors to the anti-inflammatory potential of *R. rosea* extracts [23]. These findings reinforce our observation that extracts enriched in rosavins and polyphenols, as achieved through optimized extraction conditions in this study, are associated with enhanced antioxidant and anti-inflammatory effects.

**Figure 4:**
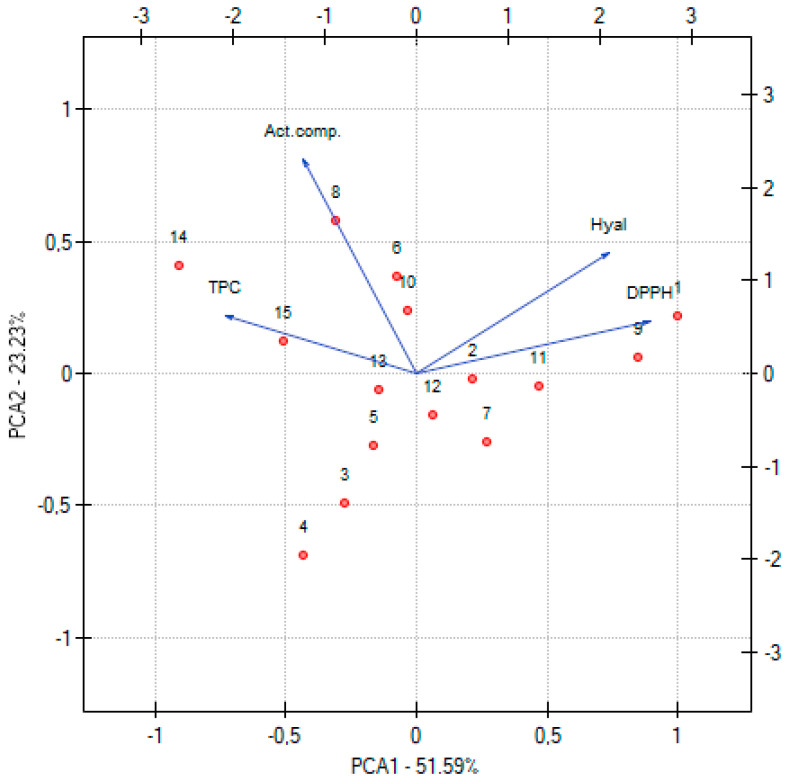
PCA for the phytochemical characterization of extracts, where the next numbers are the numbers corresponding to the extracts.

Principal Component Analysis (PCA) (Figure 4) provided valuable insights into the relationships between the phytochemical composition of the *Rhodiola rosea* extracts and their biological activities. The PCA plot demonstrated clear clustering of extracts based on the content of total polyphenols (TPC), active compounds (Act. comp.), and their antioxidant (DPPH) and anti-inflammatory (Hyal) activities, highlighting the influence of extraction parameters on extract profiles. A negative correlation between TPC and DPPH (r = −0.54) indicates that higher polyphenol content is associated with stronger antioxidant activity (lower IC_50_ values). This aligns with the role of polyphenols as potent scavengers of free radicals. A weaker negative correlation between TPC and hyaluronidase IC50 (r = −0.24) suggests a modest contribution of polyphenols to anti-inflammatory activity. The sum of active compounds (Act. comp.) showed low correlation with both DPPH (r = −0.22) and hyaluronidase (r = −0.11), implying that while these constituents contribute to bioactivity, other factors or minor compounds may also play significant roles. A positive correlation between DPPH and hyaluronidase (r = 0.62) reflects that extracts with lower antioxidant activity tended also to have weaker anti-inflammatory properties, emphasizing shared mechanisms or co-extracted bioactive components contributing to both effects.

From a practical standpoint, the PCA underscores that optimized extraction conditions enhancing TPC—particularly elevated temperature and higher methanol concentration—are key to producing extracts with superior antioxidant and anti-inflammatory properties. These findings have direct application in guiding extraction process parameters for products targeting oxidative stress and inflammation.

**Figure 5:**
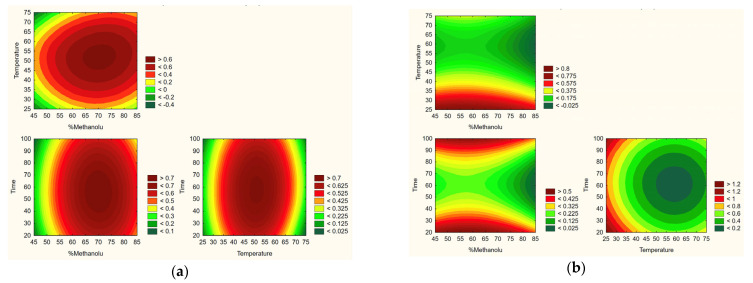
Prediction of the optimization model for obtaining extracts based on effect with positive sign-like TPC and active compounds content (**a**) and those with negative sign-like DPPH and hyaluronidase assays (**b**).

Based on the obtained research results and statistical analysis, the optimal extraction process parameters were determined: temperature of 55 °C, 75% methanol content in the extraction mixture, and extraction time of 60 min (Figure 5).

### 2.2. Obtaining Electrospun Nanofibers Containing Radiola Extract

**Figure 6:**
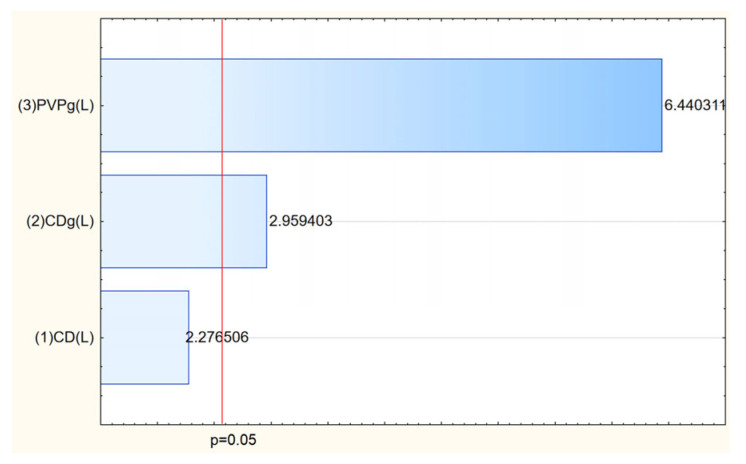
Pareto chart of the influence of input parameters on the efficiency of the process.

The next stage of the study was the preparation of electrospun systems containing *Rhodiola rosea* extract. DoE was used to assess the effect of parameters, and an experiment plan was created. The electrospinning process successfully enabled the incorporation of *Rhodiola rosea* extract into matrices, resulting in systems with tailored morphology and enhanced functional properties. The process efficiency varied depending on the composition of polyvinylpyrrolidone (PVP) and cyclodextrins (CDs), with the highest yield (71.29%) achieved for the system containing HPγCD and 3 g PVP (Table 3, Figure 6). This confirms the crucial role of both polymer concentration and cyclodextrin type in stabilizing the electrospinning process and facilitating nanofiber formation.

**Figure 7:**
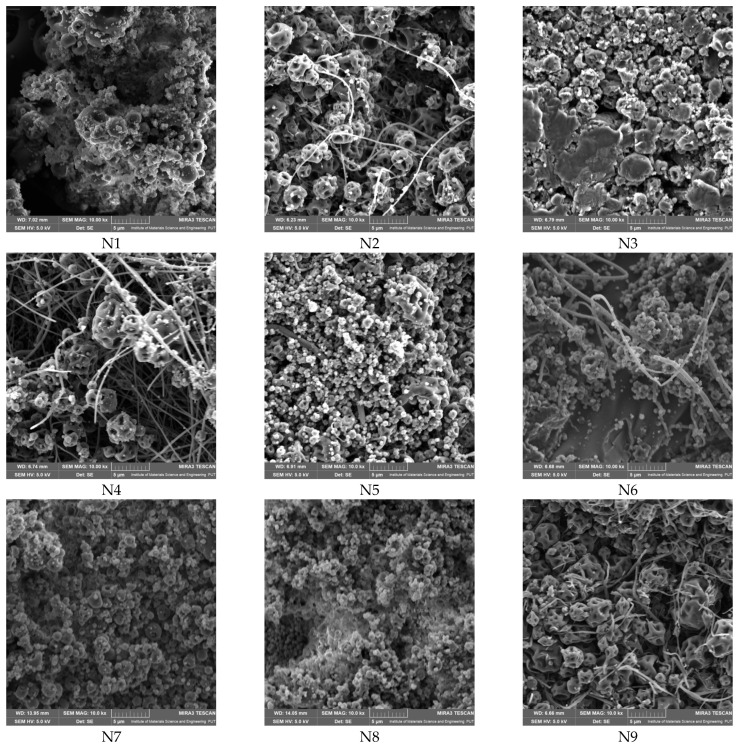
SEM images of systems N1–N9.

Scanning electron microscopy (SEM) analysis revealed that only selected compositions (notably those with balanced PVP and CD content) yielded uniform nanofibers with diameters in the nano- to submicron range (e.g., N2, N4, N5, N6, N9), whereas other systems resulted in bead formation or irregular morphologies (Figure 7, Table 4). The SEM micrographs show that, alongside nanofiber structures, there are visible irregularities such as bead formation, aggregated material, or areas of lower fiber density. In some cases, the amount of continuous nanofibers appears limited, suggesting partial rather than complete fiber formation. These findings indicate that, while electrospinning was successful in generating nanoscale structures, the resulting mats may contain microstructured regions rather than fully uniform nanofiber networks.

The superior ability of systems containing HPβCD to form uniform nanofibers, in contrast to those with HPαCD or HPγCD, which predominantly resulted in bead formation or irregular morphologies and can be explained by several physicochemical factors. HPβCD offers an optimal balance between cavity size and compatibility with both PVP and the active components of *Rhodiola rosea* extract. Its medium-sized cavity facilitates stable inclusion complex formation with bioactives such as rosavins and salidroside, promoting homogeneous dispersion of these compounds within the polymer matrix and supporting stable fiber formation. In contrast, the smaller cavity of HPαCD may inadequately accommodate these actives, leading to phase separation during electrospinning, while the larger cavity of HPγCD could form less compact or weaker complexes, compromising matrix integrity [17]. Additionally, HPβCD contributes to achieving the critical viscosity and polymer chain entanglement required for effective electrospinning [24]. The combinations containing HPαCD or HPγCD may produce solutions with suboptimal viscosity—either too low, favoring bead formation, or too high, resulting in jet instability. FTIR analyses further suggest that stronger hydrogen bonding interactions occur between PVP and HPβCD compared to the other cyclodextrins, which supports greater stability of the electrospinning jet and uniform fiber morphology. Moreover, the lower hygroscopicity of HPβCD reduces the likelihood of excess moisture interfering with jet drying, unlike HPγCD, which is more hygroscopic and thus prone to generating beads or fused structures. These findings are consistent with previous studies, in which HPβCD has been shown to enhance solubility and stabilize nanofiber formation in electrospun systems, confirming its suitability for creating uniform, bead-free nanofibers in this context.

**Figure 8:**
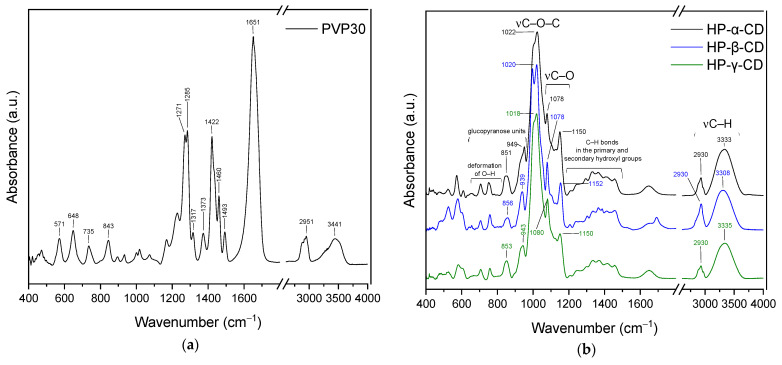
FTIR spectra of PVP (**a**) and cyclodextrins (**b**).

FTIR spectra of pure PVP K30 show characteristic absorption bands corresponding to the functional groups present in the polymer structure (Figure 8a). The band at 843 cm^−1^ is attributed vibrations of the pyrrolidone ring (cyclic –C–C– stretching mode), while peaks at 1271 cm^−1^ and 1285 cm^−1^ correspond to C–N stretching modes within the lactam ring. The absorption at 1373 cm^−1^ is assigned to CH deformation modes from –CH_2_ groups. The bands at 1422 cm^−1^ and 1460 cm^−1^ are related to CH_2_ bending vibrations. A prominent band at 1651 cm^−1^ corresponds to the C=O stretching vibration of the tertiary amide group in N-vinylpyrrolidone. In the higher wavenumber region, the band at 2951 cm^−1^ arises from C–H stretching vibrations in the polymer backbone, whereas the broad absorption around 3400 cm^−1^ is attributed to O–H stretching, indicating hydrogen bonding interactions between water molecules and the carbonyl groups of PVP, confirming its hygroscopic nature [25,26,27]. The FT-IR spectra of hydroxypropylated α-, β-, and γ-cyclodextrins (HP-α-CD, HP-β-CD, and HP-γ-CD) are presented in Figure 8b. The spectra reveal characteristic absorption bands, which are marked along with their assignments in the figure. Cyclodextrins have broad bands in the range of 3200–3600 cm^−1^ attributed to O–H stretching vibrations. This band is slightly shifted in each spectrum. Maxima is observed at about 3333 cm^−1^ (HP-α-CD), 3308 cm^−1^ (HP-β-CD), and 3335 cm^−1^ (HP-γ-CD), reflecting variations in hydrogen bonding environments due to the different cavity sizes and substitution patterns. The band near 2930 cm^−1^ in all spectra corresponds to C–H stretching vibrations of aliphatic chains. This is a typical feature of the hydroxypropyl groups. The region between 1400 and 1200 cm^−1^ shows bands arising from the bending vibrations of C–H bonds in the hydroxyl groups at primary and secondary positions. Strong bands observed around 1020–1022 cm^−1^ (C–O–C stretching) are characteristic of the ether bonds in the glucopyranose rings. Peaks observed at about 1078 cm^−1^, 1089 cm^−1^, and in the range of 1150–1152 cm^−1^, can be attributed to C–O stretching vibrations of primary and secondary alcohol groups in the cyclodextrin backbone and substituents. Whereas bands around 850–860 cm^−1^ and 940–950 cm^−1^ are associated with deformation vibrations of O–H and skeletal vibrations of the glucopyranose units, these bands further confirm the presence of cyclodextrin core structures [28,29].

**Figure 9:**
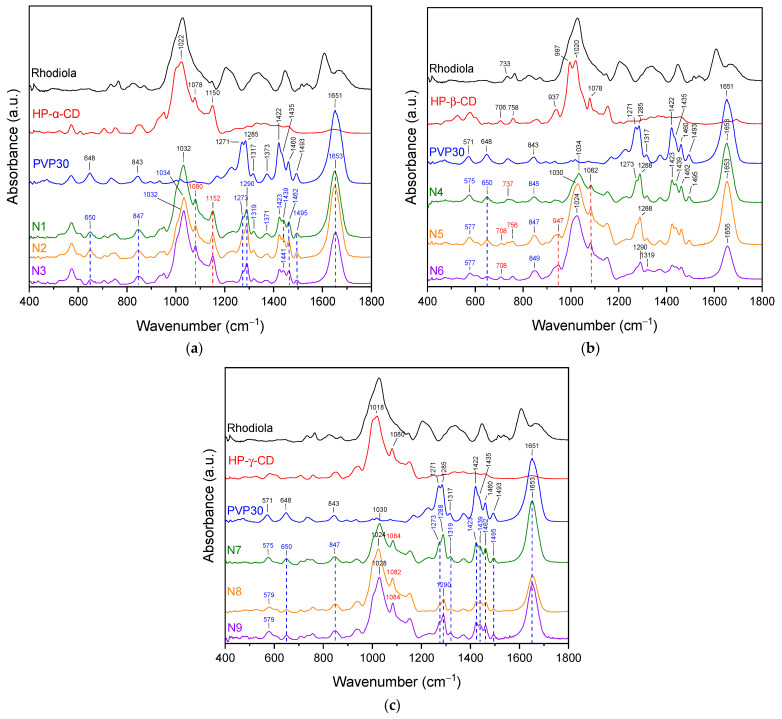
FTIR spectra of systems N1-N3 (**a**), N4-N6 (**b**), and N7-N9 (**c**).

In the spectra of N1–N3 (Figure 9a), no bands characteristic of Rhodiola are observed, confirming its complete dispersion within the PVP:HP-α-CD matrix as well as low concentration of individual compounds in the polymer mass (see Table 4). The spectra represent a superposition of PVP and HP-α-CD bands, and the changes observed in their character indicate the formation of interactions between the polymer and cyclodextrin. The main spectral changes concern bands originating from PVP. In samples N1–N3, shifts in the following bands are observed: 1651 cm^−1^→1653 cm^−1^, 1493 cm^−1^→1495 cm^−1^, 1493 cm^−1^→1462 cm^−1^, 1422 cm^−1^→1423 cm^−1^, 1373 cm^−1^→1371 cm^−1^, 1317 cm^−1^→1319 cm^−1^, 1285 cm^−1^→1290 cm^−1^, 1271 cm^−1^→1273 cm^−1^, 843 cm^−1^→847 cm^−1^, 648 cm^−1^→650 cm^−1^. The band at 1435 cm^−1^ is observed at 1439 cm^−1^ (N1 and N2) and 1441 cm^−1^ (N3). Regarding HP-α-CD, the bands originally at 1022 cm^−1^, 1078 cm^−1^, and 1150 cm^−1^ are shifted to higher wavenumbers in N1–N3 and are observed at 1032 cm^−1^, 1080 cm^−1^, and 1152 cm^−1^, respectively. The band which shifted from 843 cm^−1^ to 847 cm^−1^, which was attributed to pyrrolidone ring vibrations (cyclic –C–C– stretching mode), indicates alterations in the ring environment [27]. This may be related to interactions with the cyclodextrin cavity. Changes observed in the bands corresponding to C–N stretching within the lactam ring (1271 cm^−1^ and 1285 cm^−1^) may suggest the formation of hydrogen bonds or spatial rearrangement within the ternary system. Shifts in the CH_2_ deformation band (1373 cm^−1^) and CH_2_ bending bands (1422 cm^−1^ and 1460 cm^−1^) indicate conformational changes or altered polymer chain mobility resulting from complex formation. Differences in the shifts of the 1435 cm^−1^ band in samples N1–N3 suggest variations in the strength of interactions. Additional evidence for PVP–cyclodextrin interactions is provided by the shifts observed in bands related to C–O–C and C–O stretching vibrations (1022 cm^−1^, 1078 cm^−1^, and 1150 cm^−1^) and slight shift of the band observed in PVP at 1651 cm^−1^ (the C=O stretching vibration of the tertiary amide group in PVP). The band which shifted from 1651 cm^−1^ to 1653 cm^−1^ in the N1–N3 spectra suggest formation of hydrogen bonds interactions between the carbonyl groups of PVP and the hydroxyl groups of HP-α-CD. In the spectra of samples N4–N6 (Figure 9b), analogous shifts to those observed in N1–N3 are present. The characteristic bands of PVP at 571, 648, 843, 1271, 1285, 1317, 1422, 1435, 1460, 1493, and 1651 cm^−1^ are shifted to higher wavenumbers, indicating changes in the local environment of the polymer chains due to interactions with HP-β-cyclodextrin. Regarding HP-β-cyclodextrin, shifts are observed in the bands at 937 cm^−1^, 1020 cm^−1^, and 1078 cm^−1^. The band at 1020 cm^−1^ corresponds to C–O–C stretching vibrations characteristic of ether linkages in the glucopyranose units, while the bands at 937 cm^−1^ and 1078 cm^−1^ are attributed to C–O stretching vibrations of primary and secondary hydroxyl groups. The observed shifts of these bands to higher wavenumbers suggest the involvement of cyclodextrin hydroxyl groups in hydrogen bonding, likely with the carbonyl or amide groups of PVP. These spectral changes confirm the formation of intermolecular interactions, most likely hydrogen bonds, between PVP and HP-β-cyclodextrin within the ternary system. In the spectra of samples N7–N9 (Figure 9c), similar spectral shifts are observed as in the previous systems, further indicating consistent interactions within the ternary matrices. The characteristic PVP bands at 571, 648, 843, 1271, 1285, 1317, 1422, 1435, 1460, 1493, and 1651 cm^−1^ are all shifted to higher wavenumbers in N7–N9. Also in this case, these shifts are indicative of non-covalent interactions, most likely hydrogen bonding, which can influence the electron density and vibrational behavior of functional groups such as the carbonyl, lactam, and CH_2_ units within PVP. Whereas for HP-γ-cyclodextrin, shifts are observed for bands at 1018 cm^−1^ and 1080 cm^−1^. The band at 1018 cm^−1^ is associated with C–O–C stretching vibrations in the ether linkages of the glucopyranose rings, while the 1080 cm^−1^ band corresponds to C–O stretching vibrations of hydroxyl groups. The shift of these bands to higher wavenumbers indicates changes in the vibrational environment of the cyclodextrin, most likely due to hydrogen bonding interactions between the hydroxyl groups of HP-γ-CD and the polar functional groups of PVP.

Confirmed hydrogen bonding contribute to the stabilization of the ternary complexes and further support the successful molecular dispersion of Rhodiola within the polymer-cyclodextrin matrix. The absence of Rhodiola-specific bands across all spectra confirms its complete incorporation and homogeneous distribution at the molecular level.

**Figure 10:**
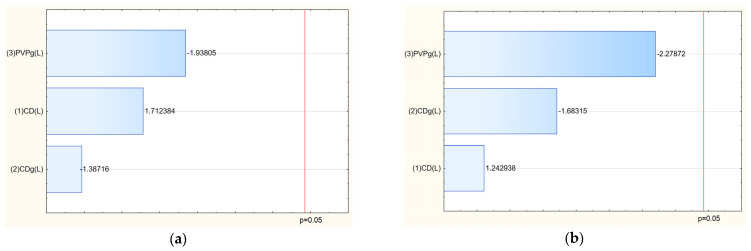
Pareto chart of the influence of input parameters on the salidroside (**a**) and rosarin (**b**) content.

After confirming the structure of nanofibers, the next stage was to study the influence of input parameters on the properties of the obtained nanofibers. The analysis of salidroside and rosarin content in the obtained nanofiber systems revealed that the concentration of both active compounds was influenced by the composition of the electrospun matrix (Table 5). As shown in the Pareto plots, a clear trend was observed: increasing the proportion of PVP in the formulation led to a decrease in the content of salidroside and rosarin in the final nanofiber systems (Figure 10). This inverse relationship suggests that higher amounts of PVP, while beneficial for fiber formation and mechanical stability, may dilute the relative concentration of active compounds in the matrix or hinder their efficient incorporation during the electrospinning process. This phenomenon can be attributed to several factors. First, excessive PVP may dominate the matrix structure, limiting the ability of cyclodextrins to effectively complex and stabilize the active compounds, thereby reducing their entrapment efficiency. Second, the high polymer load may lead to phase separation or uneven distribution of the extract components within the nanofiber structure during the rapid solvent evaporation that occurs in electrospinning. Finally, as PVP is a hydrophilic carrier, its excessive presence might enhance the solubility and partial loss of active compounds during processing or sample preparation for analysis [30].

**Figure 11:**
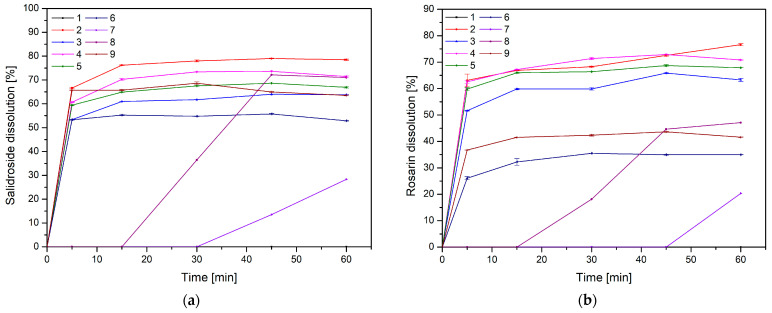
Release profiles of salidroside (**a**) and rosarin (**b**).

**Figure 12:**
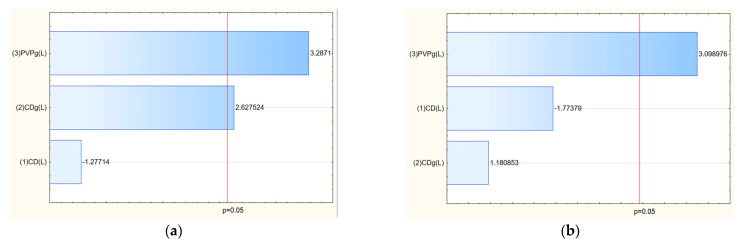
Pareto chart illustrating the influence of input parameters on the salidroside (**a**) and rosarin (**b**) release.

Then, a release study was performed for salidroside and rosavins contained in the nanofibers (Figure 11). Data on the percentage release of salidroside and rosarin from the produced nanofibers after 5 min were used to develop a statistical model. An increase in the release rate can be observed at 5 min for nanofibers 2–6 and 9, which remains at a constant level in the following minutes. On the other hand, systems 1, 7, and 8 were characterized by much worse solubility, for which no release was observed at 5 min. The release profiles of salidroside and rosarin from the produced nanofiber systems showed distinct patterns influenced by the composition of the electrospun matrix. Analysis of the Pareto plots revealed that increasing the PVP content in the nanofibers significantly accelerated the release rate of both salidroside and rosarin (Figure 12). This can be explained by the hydrophilic nature of PVP, which enhances water uptake into the fiber matrix, promotes swelling, and facilitates faster dissolution and diffusion of the incorporated active compounds [13]. A higher proportion of PVP creates a more permeable network upon contact with aqueous environments, reducing the diffusion barrier for salidroside and rosarin.

The release kinetics of salidroside from the electrospun nanofiber systems were analyzed using four mathematical models commonly applied to controlled release systems: zero-order, first-order, Higuchi, and Korsmeyer–Peppas (Table 6). The evaluation of the coefficient of determination (R^2^) for each model allowed the identification of the most appropriate kinetic profile for each formulation. In most systems (N2–N6, N8, N9), the Higuchi model provided the best fit to the experimental data, indicating that salidroside release was predominantly governed by diffusion from the polymeric matrix. This is consistent with the structural characteristics of the nanofibers, where the drug is molecularly dispersed within the hydrophilic matrix formed by PVP and cyclodextrin. Notably, system N8 demonstrated a high degree of fit not only to the Higuchi model but also to the zero-order model, suggesting a near-constant release rate over time, likely due to an optimal balance between polymer composition, drug loading, and matrix porosity. Furthermore, the Korsmeyer–Peppas model provided additional insight into the mechanism of salidroside release. The diffusion exponent *n* for all analyzable systems fell within the range of 0.45 to 0.89, indicative of non-Fickian or anomalous transport. This suggests that both diffusion of the drug and relaxation of the polymer chains contributed to the release mechanism. Such behavior is typical for electrospun fiber systems where the morphology, swelling, and hydration dynamics play significant roles in drug mobility.

To complement these findings, the Weibull model was also applied, offering a more flexible empirical approach to capture the diverse release profiles observed. Formulations N2–N5 displayed β values below 1, consistent with diffusion-controlled kinetics, while N7 and N8 exhibited β values greater than 1, indicating a sigmoidal release pattern with an initial lag phase followed by accelerated release. This may be attributed to matrix restructuring or burst release phenomena. The incorporation of the Weibull model thus provided additional resolution in characterizing and differentiating the release mechanisms. Collectively, these modeling approaches confirm that salidroside release from the nanofiber systems is governed by a combination of matrix hydration, diffusion, and polymer relaxation. The formulation parameters, particularly the type and concentration of PVP and cyclodextrin, emerge as critical factors influencing release behavior and should be carefully optimized to enhance the bioavailability of *Rhodiola rosea* bioactives in future delivery systems.

Furthermore, the type of cyclodextrin used in the nanofiber systems had a differentiated impact on the release of each active compound. For salidroside, the use of HPγCD resulted in the most pronounced improvement in solubilization and release. This is likely due to the larger cavity size of HPγCD, which better accommodates the relatively small and polar salidroside molecule, forming more stable and effective inclusion complexes that enhance its solubility and subsequent release. In contrast, for rosarin, nanofibers containing HPαCD showed the most efficient release. Although HPαCD has a smaller cavity, this may provide a better fit for the rosarin molecule’s specific structure, favoring stronger host–guest interactions and facilitating its release from the nanofiber matrix.

The permeability study of salidroside and rosarin aimed to evaluate how effectively these active compounds, incorporated into electrospun nanofiber systems, could penetrate artificial biological membranes, simulating gastrointestinal absorption. The results demonstrated a substantial enhancement in the apparent permeability coefficients (P_app_) of both salidroside and rosarin when delivered via nanofiber systems, compared to the crude extract (Table 7). Systems with faster release (due to optimal PVP and cyclodextrin balance) provided higher local concentrations of actives at the membrane interface, thereby driving passive diffusion. The nanofiber matrix not only facilitated this rapid release but also likely improved wettability and reduced crystallinity of the actives, both factors contributing to higher permeability.

**Figure 13:**
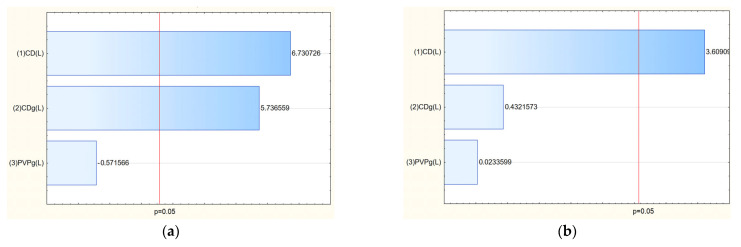
Pareto chart of the influence of input parameters on the salidroside (**a**) and rosarin (**b**) permeability.

The most important factor influencing the permeation of active compounds turned out to be the selection of the appropriate cyclodextrin (Figure 13), and in the case of salidroside, its amount was also important. The highest permeability through artificial biological membranes for rosarin was observed in nanofibers containing HPβCD and HPγCD in their structure. In the case of salidroside, the most effective permeation was observed when using HPγCD.

**Figure 14:**
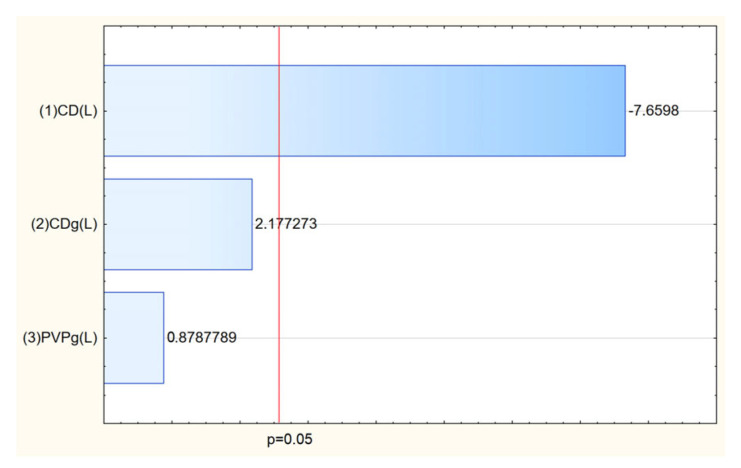
Pareto chart of the influence of input parameters on mucoadhesive properties.

The mucoadhesive properties of the electrospun nanofiber systems were evaluated to assess their ability to remain at the site of application, which is critical for ensuring prolonged contact time and improved local delivery of active compounds (Table 8). The mucoadhesiveness was quantified using a viscometric method, where the interaction between the nanofiber systems and mucin was measured. The key factor influencing mucoadhesion was the type of cyclodextrin incorporated into the nanofiber matrix (Figure 14). The presence of HPαCD in the formulation was associated with superior mucoadhesive properties. This is likely because HPαCD, due to its smaller cavity and specific interaction potential, promoted stronger binding interactions with mucin or contributed to a more cohesive matrix that enhanced mucoadhesion. Systems containing HPβCD and HPγCD generally exhibited lower mucoadhesion, possibly due to weaker interactions with mucin or different structural characteristics of the formed fibers.

**Figure 15:**
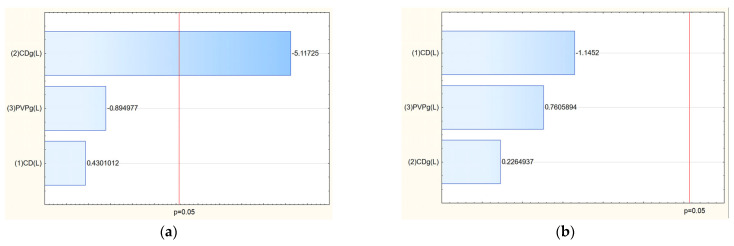
Pareto chart of the influence of input parameters on antioxidant (**a**) and anti-inflammatory (**b**) properties of systems.

The antioxidant and anti-inflammatory activities of the electrospun nanofiber systems were evaluated using the DPPH assay (to measure free radical scavenging ability) and the hyaluronidase inhibition assay (to assess anti-inflammatory potential) (Table 9). The results demonstrated that incorporating *Rhodiola rosea* extract into nanofiber matrices notably enhanced antioxidant activity while having a varied impact on anti-inflammatory properties compared to the raw extract. The amount and type of cyclodextrin were identified as key factors influencing antioxidant activity, with systems containing higher amounts of cyclodextrin generally showing stronger antioxidant performance. This likely stems from better dispersion and stabilization of the active compounds within the nanofiber matrix, enhancing their ability to scavenge free radicals (Figure 15).

Interestingly, while electrospinning generally improved antioxidant properties, the anti-inflammatory activity of many nanofiber systems (N2–N8) was lower than that of the original extract. This suggests that the process or matrix composition may have altered the accessibility or efficacy of certain bioactive compounds responsible for enzyme inhibition.

**Figure 16:**
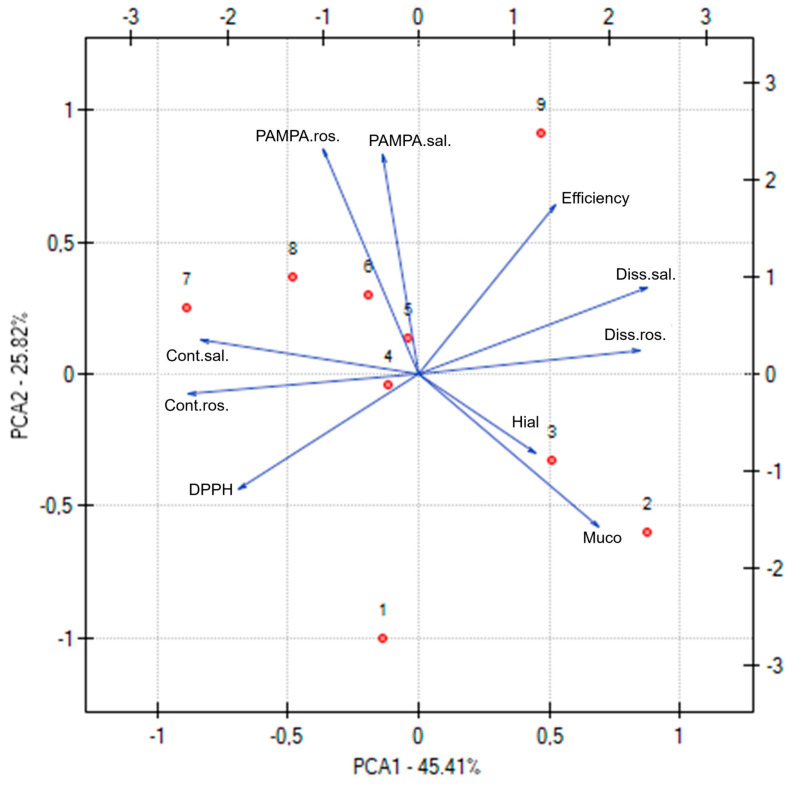
PCA for the characterization of electrospun systems, where the next numbers are the numbers corresponding to the systems.

The Principal Component Analysis (PCA), presented in Figure 16, was performed to explore and visualize the relationships between various functional, technological, and compositional parameters of the electrospun nanofiber systems. Each point on the PCA plot corresponds to one of the nanofiber systems (N1 to N9), allowing a comprehensive comparison of their profiles. The PCA revealed distinct clustering patterns among the nanofiber formulations, highlighting how variations in composition (e.g., type and amount of cyclodextrin and PVP) influence the overall properties. Systems with similar functional characteristics, such as antioxidant activity (DPPH), anti-inflammatory activity (hyaluronidase inhibition), permeability coefficients (PAMPA rosarin and salidroside), dissolution rates (salidroside and rosarin release), and mucoadhesiveness, tended to group together. Nanofiber systems with higher PVP content generally clustered in regions associated with improved dissolution rates and permeability, reflecting the role of PVP in promoting hydrophilicity and release. Formulations containing HPαCD were associated with stronger mucoadhesive properties, as seen in their distinct grouping on the plot. Systems combining HPβCD and an intermediate PVP level (e.g., N5) occupied a favorable position in the PCA space, balancing high active compound content, effective release, permeability, and bioactivity.

Moreover, the correlation matrix included in the PCA analysis provides detailed insight into how individual properties of the electrospun nanofiber systems are related to each other. This matrix displays correlation coefficients (r values) that range from −1 to 1, indicating the strength and direction of linear relationships between pairs of variables. A very high correlation was observed between salidroside content and rosarin content (r = 0.93), reflecting that formulations effective at incorporating salidroside also tended to load rosarin efficiently. Dissolution of salidroside and rosarin were strongly correlated (r = 0.91), indicating that formulations promoting rapid release of salidroside similarly enhanced rosarin release. A negative correlation between DPPH and salidroside content (r = −0.57) and between DPPH and rosarin content (r = −0.70) implies that higher active compound content led to stronger antioxidant activity (lower IC_50_ values). DPPH was also negatively correlated with dissolution rates (e.g., r = −0.68 with salidroside dissolution), suggesting that faster release supported better antioxidant performance. Both PAMPA salidroside permeability and PAMPA rosarin permeability showed negative correlations with mucoadhesive properties (e.g., PAMPA rosarin r = −0.76 with mucoadhesion), indicating that formulations with higher mucoadhesion tended to exhibit lower permeability—likely due to stronger interactions at the mucosal interface reducing diffusion.

**Figure 17:**
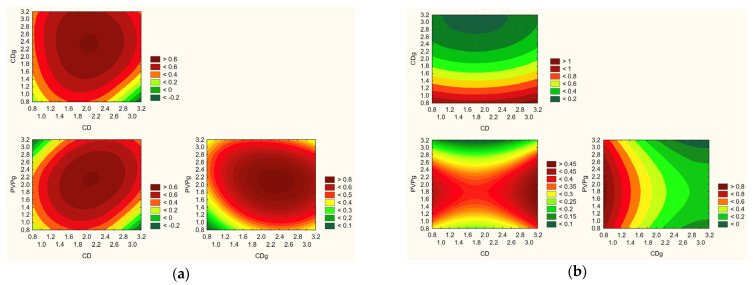
Prediction of the optimization model for obtaining systems for effect with positive sign: efficiency, content, release and permeability of active components, and mucoadhesion properties (**a**) and with negative sign; antioxidant and anti-inflammatory activity (**b**).

The predictive modeling of nanofiber system properties, as illustrated in Figure 17, enabled identification of the optimal composition for achieving a balanced and favorable profile of functional and technological characteristics. The model integrated data from the Design of Experiments (DoE) approach, considering multiple key parameters: active compound content, release rate, permeability, mucoadhesion, antioxidant activity, and anti-inflammatory activity. According to the model, the most favorable nanofiber formulation was obtained with a composition containing 3 g of hydroxypropyl-β-cyclodextrin (HPβCD) and 2.5 g of polyvinylpyrrolidone (PVP).

This specific ratio provided the best compromise between high loading of active compounds, effective and rapid release, enhanced permeability across biological membranes, strong antioxidant activity, and acceptable anti-inflammatory properties. Additionally, this composition supported the formation of uniform nanofibers with desirable morphology and mucoadhesive characteristics, essential for stability and prolonged retention at the site of application. The selection of HPβCD at 3 g likely contributed to optimal inclusion complex formation with the *Rhodiola rosea* actives, enhancing their solubility, stabilization, and release profile. The 2.5 g PVP level provided sufficient hydrophilicity to promote rapid dissolution while maintaining fiber integrity and mechanical stability during processing and use. The predictive model highlighted the ideal formulation for maximizing the therapeutic and functional potential of the electrospun nanofiber systems for bioactive delivery.

The present study provides a comprehensive in vitro characterization of electrospun nanofiber systems containing *Rhodiola rosea* extract, demonstrating significant improvements in dissolution rate, membrane permeability, and antioxidant activity of key bioactive compounds. These findings offer valuable insights into the potential of cyclodextrin/PVP-based nanofibers as a promising delivery platform. However, it should be noted that in vitro results, while encouraging, may not fully reflect in vivo conditions. Factors such as metabolism, systemic absorption, and pharmacokinetics, particularly relevant for compounds like salidroside, could influence bioavailability and therapeutic outcomes. Similarly, while the in vitro mucoadhesion assay provided useful comparative data across formulations, its direct relevance to mucosal retention in the human body remains uncertain. Future studies should therefore include in vivo validation, such as pharmacokinetic profiling or animal models, and consider the use of clinically relevant mucoadhesion models to confirm the translational potential of these promising systems.

## 3. Materials and Methods

### 3.1. Plant Material

The plant material, root of *Rhodiola rosea*, was purchased as a commercially available material from NANGA; country of origin: China; best before date March 2025; batch number: 5544P.

### 3.2. Chemicals and Reagents

Salidroside (phyproof^®^ Reference Substances), p-tyrosol (98%), rosavin (phyproof^®^ Reference Substances), rosarin (phyproof^®^ Reference Substances), and rosin (phyproof^®^ Reference Substances) were obtained from Sigma-Aldrich (Poznan, Poland). Carriers such as 2-Hydroxypropyl)-α-cyclodextrin (HPαCD) average Mw ~1180 g/mol, (2-Hydroxypropyl)-β-cyclodextrin (HPβCD) average Mw ~1460 g/mol, 2-Hydroxypropyl)-γ-cyclodextrin (HPγCD) average Mw ~1580 g/mol, and polyvinylpyrrolidone (PVP) K30 average Mw ~40,000 g/mol were supplied by Sigma-Aldrich (Poznan, Poland). Sigma-Aldrich (Poznan, Poland) provided reagents like Folin–Ciocalteu reagent, 2.2-Diphenyl-1-picrylhydrazyl (DPPH), bovine serum, hexadecyltrimethylammonium bromide (CTAB), and hyaluronic acid (HA) with Mw 1.5 MDa for activity assays; KCl, NaCl, K_2_HPO_4_, MgCl_2_, CaCl_2_, and xylitol for dissolution studies; and mucin from the porcine stomach for a mucoadhesive assay. Prisma™ HT buffer, Acceptor Sink Buffer, and GIT lipid solution were obtained from Pion Inc. (Billerica, MA, USA), whereas HPLC grade acetonitrile and water were obtained from Merck (Darmstadt, Germany). High-quality pure water and ultra-high-quality pure water were prepared using a Direct-Q 3 UV Merck Millipore purification system (Burlington, MA, USA).

### 3.3. Optimization of the Radiola Extract Extraction Process and Investigation of Its Biological Activity

#### 3.3.1. Optimization of the Radiola Extract Extraction Process

The Design of Experiments (DoE) method was used to generate a 3-level Box–Behnken plan. The extraction mixture’s temperature, composition, and processing time were selected as independent variables (Table 10).

The following metrics were selected to evaluate the effectiveness of the extraction process: total phenolic compound content, active components content, antioxidant activity (DPPH scavenging assay), and anti-inflammatory activities expressed as the suppression of hyaluronidase activity.

#### 3.3.2. Investigation of Nanofibers Phytochemical and Biological Activities

The total content of phenolic components (TPC) was determined using the method described previously [16]. Briefly, each vial was filled with 200 µL of distilled water, 15 µL of Folin–Ciocalteu reagent, 60 µL of 20% sodium carbonate solution, and 25 µL of extract or gallic acid solution. After five minutes of 600 rpm shaking, the plate was left to stand at room temperature for a further twenty-five minutes in the dark. At 760 nm, the absorbance was measured using a Multiskan GO 1510, manufactured by Thermo Fisher Scientific in Vantaa, Finland. The phenolic content of the extracts was measured in milligrams of gallic acid equivalents (GAE) per gram of the plant material. Each experiment was repeated nine times.

A high-performance liquid chromatography technique has been developed to measure the concentration of five components (salidroside, p-tyrosol, rosavin, rosarin, rosin) at the same time. A LiChrospher RP-18 column (5 μm, 250 mm × 4 mm) (Merck, Darmstadt, Germany) served as the stationary phase. With a continuous mobile phase flow of 1.2 mL/min, the mobile phase consisted of formic acid 0.1% (A) and acetonitrile (B) in a gradient flow: 0–10 min, 4% B; 10–20 min, 13% B; 20–30 min, 15% B; 30–33 min, 20% B; 33–38 min, 25% B; 38–60 min, 30% B. The temperature of the column was set at 30 °C. It was successfully detected at *λ*_max_ = 275 nm. The linearity and limits of detection and quantification were assessed in accordance with the International Conference on Harmonization Guideline (ICH Q2). Each experiment was repeated six times.

Antioxidant activity was determined using an assay with 2.2-Diphenyl-1-picrylhydrazyl (DPPH), as described previously [31]. During a 30-min dark incubation period, 25 μL of extract was constantly mixed with 175 μL of a 0.2 mmol/L DPPH solution. The absorbance was measured at 517 nm in relation to the blank sample, which was made up of 25 μL of the extraction mixture and 175 μL of methanol (Multiskan GO 1510, Thermo Fisher Scientific, Vantaa, Finland). Each experiment was repeated nine times.

The previously established turbidimetric approach was used to determine the hyaluronidase inhibition process [31]. Acetate buffer (pH 4.5; 15 µL), hyaluronidase enzyme (30 U/mL; 25 µL), extract (10 µL), and incubation buffer (50 mM. pH 7.0 with 77 mM NaCl and 1 mg/mL of bovine albumin; 25 µL) were combined to create the sample. After 10 min of incubation at 37 °C, a hyaluronic acid solution (0.3 mg/mL; 25 µL) was added, and the combination was incubated for an additional 45 min. After adding 200 µL of CTAB and waiting 10 min at room temperature, the turbidity was measured at 600 nm using a Multiskan GO 1510 (Thermo Fisher Scientific, Vantaa, Finland). Each experiment was repeated nine times.

### 3.4. Obtaining Electrospun Nanofibers Containing Radiola Extract

The NS + NanoSpinner Plus Electrospinning Equipment (Inovenso Ltd., Istanbul, Turkey) was used to perform the electrospinning procedure. Cyclodextrin type, cyclodextrin, and PVP composition used to create the nanofibers was selected based on Design of Experiment (DoE) data and the 3^2^ full factorial design experimental plan (Table 11). All ingredients, according to Table 11, together with 5 mL of optimized extract, were added to 20 mL of methanol and mixed on a magnetic stirrer for 30 min. The high voltage was set at 27 kV, the solution flow rate was 2 mL/h, and the syringe with a 22G needle was placed 12 cm away from the revolving collector wrapped in aluminum foil. The experiments were carried out at 25 °C and with a maximum humidity of 40%.

The parameters used to quantify the DoE process response were efficiency, content of active ingredients, release of active compounds and their penetration, mucoadhesive properties, and biological activity.

### 3.5. Characterization of the Electrospun Nanofibers

#### 3.5.1. Scanning Electron Microscopy (SEM)

SEM was used to view the nanofiber’s surface morphology. After being coated with gold-palladium sputter, the nanofibers were inspected using a Quanta 250 FEG (FEI Company, Eindhoven, The Netherlands), which is an FE scanning electron microscope. The ImageJ software (https://imagej.net/ij/download.html (accessed on 03 July 2025)) was used to measure the nanofibers’ diameter.

#### 3.5.2. Fourier Transform Infrared Spectroscopy (FTIR)

An IRTracer-100 (Shimadzu, Kyoto, Japan) spectrophotometer and LabSolutions IR software (version 1.86 SP2, Shimadzu, Kyoto, Japan) were used to obtain FTIR spectra in absorbance mode, covering 400 to 4000 cm^−1^. The spectrometer was configured with 400 scans, Happ–Genzel apodization, and a resolution of 4 cm^−1^.

### 3.6. Characterization of Electrospun Nanofiber’s Functionality

#### 3.6.1. Salidroside and Rosarin Release and Permeability Assays

The rate of dissolution of salidroside and rosarin integrated into the nanofibers, and its penetration through the membrane systems that mimic the walls of the gastrointestinal tract following the release from the nanofibers, was assessed using the chromatographic technique. After active components were incorporated into the structure of the nanofibers, their antioxidant capability was evaluated using the spectroscopic approach.

An Agilent 708-DS dissolving instrument (Agilent Technologies, Santa Clara, CA, USA) was used to conduct dissolve tests on electrospun nanofibers. A conventional basket method was used to stir at 37 ± 0.5 °C and 50 rpm. Nanofibers were combined with 30 mL of artificial saliva (pH 6.8), potassium chloride 1.20 g, sodium chloride 0.85 g, dipotassium hydrogen orthophosphate 0.35 g, calcium chloride 0.20 g, xylitol 20.0 g, and water up to 1 L. The pH was lowered to 6.8 using 1 M HCl. Instead of taking liquid samples at predetermined times, an equivalent volume of a temperature-stabilized medium was utilized. A nylon membrane filter with a mesh size of 0.45 µm was used to filter the samples. The amounts of salidroside and rosarin in the filtrated acceptor solutions were determined using the previously described HPLC procedure. Each experiment was repeated three times.

The permeability of the salidroside and rosarin encapsulated in the nanofibers across synthetic biological membranes was investigated using the Pion Inc. parallel artificial membrane permeability assay (PAMPA^TM^) gastrointestinal tract (GIT) assay. The nanofibers were dissolved using donor solutions, which were synthetic saliva solutions with a pH of 6.8. The pH 7.4 Acceptor Prisma buffer was added to the acceptor plates. Following plate assembly, the plates were continuously stirred at 50 rpm for 15 min at 37 °C. At least three repetitions of each experiment were conducted. The previously mentioned HPLC method was used to determine the level of performance for the salidroside and rosarin. Each experiment was repeated three times.

The apparent permeability coefficients (*P_app_*) were calculated using the following equation:

$$P_{app}=\frac{-ln\left(1\;-\;\frac{C_{A}}{C_{equilibrium}}\right)}{S\;\times \;\left(\frac{1}{V_{D}}\;+\;\frac{1}{V_{A}}\right)\times t}$$

where *V_D_*—donor volume, *V_A_*—acceptor volume, *C_equilibrium_*—equilibrium concentration $C_{equilibrium}=\frac{C_{D}\;\times {\;V}_{D}\;+\;C_{A}\;\times {\;V}_{A}}{V_{D}\;+\;V_{A}}$, *C_D_*—donor concentration, *C_A_*—acceptor concentration, *S*—membrane area, and *t*—incubation time (in seconds).

#### 3.6.2. The Nanofibers’ Activity

The antioxidant activity of the electrospun nanofibers was checked using the DPPH assay, and the anti-inflammatory activity was determined as the ability to inhibit the activity of the hyaluronidase enzyme, as described in Section 3.3. Each experiment was repeated nine times.

#### 3.6.3. Mucoadhesive Properties

The strength of the binding between mucin and the polymers for bioadhesion was evaluated using a viscometric technique. The assessment was conducted using the previously mentioned technique [16]. The enhanced intermolecular frictional force per unit area, or force of bioadhesion *F*, was calculated as follows:

*F* = (*η_t_* − *η_m_* − *η_p_*) × *σ*

where *η_t_* is the nanofibers’ viscosity coefficient, *η_m_* is mucin’s viscosity coefficient, *η_p_* is PVP/HPxCD’s viscosity coefficient, and *σ* is the rate of shear per second. Each experiment was repeated three times.

### 3.7. Statistical Analysis

Statistica 13.3 software was used to perform statistical analyses and Design of Experiment (DoE) research. To ascertain whether the data were normal, the Shapiro–Wilk test was employed. The differences between the mean values were examined using the ANOVA test and Duncan’s post hoc tests for multiple comparisons. At *p* < 0.05, group differences were considered significant. PQStat Software version 1.8.4.142 (2022) was utilized to evaluate correlations utilizing principal component analysis (PCA).

## 4. Conclusions

This study demonstrated the successful development of electrospun nanofiber systems containing *Rhodiola rosea* extract, using polyvinylpyrrolidone (PVP) and hydroxypropyl-cyclodextrins (HPαCD, HPβCD, HPγCD) as carriers. Through the application of a design of experiments (DoE) approach, it was possible to identify formulations that provided enhanced technological and functional properties compared to the crude extract. The nanofibers significantly improved the dissolution rate, membrane permeability, and antioxidant activity of salidroside and rosarin, while also offering tailored mucoadhesive properties. The composition containing 5 mL of optimized extract, 3 g of HPβCD and 2.5 g of PVP, in 20 mL of methanol, emerged as the most favorable, delivering a balanced profile of high active compound loading, rapid and efficient release, improved permeability, potent antioxidant activity, and acceptable anti-inflammatory effects. The results highlighted the critical role of both cyclodextrin type and polymer concentration in modulating the structure, bioactivity, and technological quality of the nanofibers. Principal component analysis confirmed that system composition was a key determinant of the relationships between release, permeability, mucoadhesion, and biological activity. Future research should focus on scaling up the electrospinning process, exploring the incorporation of additional plant bioactives for multifunctional delivery, assessing the long-term stability of the nanofibers, and developing specific dosage forms such as orodispersible films or buccal patches represent important steps toward translating these findings into practical pharmaceutical or nutraceutical applications.

## Figures and Tables

**Table 1:** TPC and content of active compounds in the *Rhodiola rosea* extract.

No.	TPC [mg GAE/g Plant Material]	Salidroside Content [mg/g Plant Material]	p-tyrosol Content [mg/g Plant Material]	Rosavin Content [mg/g Plant Material]	Rosarin Content [mg/g Plant Material]	Rosin Content [mg/g Plant Material]	Sum of Active Components [mg/g Plant Material]
E1	54.89 ± 1.93 ^h^	6.53 ± 2.96	7.36 ± 3.99	0.66 ± 0.42	0.63 ± 0.16	0.66 ± 0.42	29.09
E2	56.84 ± 1.12 ^g,h^	9.51 ± 1.60	5.32 ± 0.10	0.31 ± 0.05	0.15 ± 0.06	0.31 ± 0.05	34.27
E3	73.11 ± 7.17 ^c,d,e^	5.42 ± 3.24	6.40 ± 3.84	0.47 ± 0.29	0.38 ± 0.21	0.47 ± 0.29	24.38
E4	73.46 ± 3.08 ^c,d,e^	5.11 ± 3.51	1.70 ± 0.10	0.47 ± 0.09	0.50 ± 0.05	0.47 ± 0.10	23.20
E5	74.27 ± 7.26 ^c,d,e^	6.44 ± 4.64	6.31 ± 4.46	0.78 ± 0.73	0.38 ± 0.35	0.78 ± 0.73	27.15
E6	68.70 ± 2.86 ^d,e,f^	8.85 ± 0.02	6.35 ± 0.11	0.45 ± 0.07	0.13 ± 0.05	0.45 ± 0.07	37.38
E7	51.69 ± 1.97 ^h^	6.57 ± 0.05	8.63 ± 0.11	0.92 ± 0.03	0.50 ± 0.35	0.92 ± 0.03	28.25
E8	77.09 ± 4.37 ^b,c,d^	9.78 ± 0.10	7.87 ± 0.08	1.19 ± 0.06	0.74 ± 0.03	1.19 ± 0.06	42.21
E9	65.65 ± 4.64 ^e,f,g^	2.49 ± 0.05	2.31 ± 0.16	0.77 ± 0.11	0.26 ± 0.12	0.77 ± 0.11	24.06
E10	84.80 ± 3.95 ^a,b^	7.23 ± 0.57	3.62 ± 0.02	0.64 ± 0.18	0.38 ± 0.21	0.64 ± 0.18	31.32
E11	61.41 ± 5.71 ^f,g,h^	6.43 ± 1.42	8.28 ± 1.90	1.21 ± 0.53	0.47 ± 0.26	1.21 ± 0.53	29.32
E12	86.00 ± 9.27 ^a,b^	4.93 ± 0.02	2.97 ± 0.05	0.53 ± 0.22	0.10 ± 0.01	0.52 ± 0.22	23.49
E13	68.50 ± 8.95 ^d,e,f^	6.52 ± 1.14	8.00 ± 1.66	1.18 ± 0.33	0.60 ± 0.17	1.18 ± 0.33	32.77
E14	94.04 ± 3.93 ^a^	8.09 ± 0.09	10.17 ± 0.23	1.56 ± 0.01	0.76 ± 0.12	1.56 ± 0.01	41.14
E15	81.27 ± 6.44 ^b,c^	7.30 ± 1.11	9.09 ± 1.54	1.37 ± 0.27	0.68 ± 0.11	1.37 ± 0.27	35.93

Mean values within a column with the same letter are not significantly different at *p* = 0.05 using Duncan’s test.

**Table 2:** Biological activity of *Rhodiola rosea* extracts.

No.	Antioxidant Activity IC_50_ [μg/mL]	Anti-inflammatory Activity IC_50_ [mg/mL]
E1	498.46 ± 22.73 ^i^	5.29 ± 0.43 ^f,g^
E2	424.30 ± 18.51 ^g^	2.35 ± 0.20 ^a,b^
E3	257.26 ± 9.66 ^a,b^	2.55 ± 0.30 ^a,b,c^
E4	243.54 ± 17.58 ^a^	1.67 ± 0.35 ^a^
E5	302.14 ± 20.12 ^c^	2.86 ± 0.42 ^b,c,d^
E6	285.04 ± 11.22 ^b,c^	4.31 ± 0.61 ^e,f^
E7	305.16 ± 17.34 ^c,d^	3.56 ± 0.50 ^c,d,e^
E8	314.49 ± 21.98 ^c,d^	3.64 ± 0.45 ^c,d,e^
E9	448.29 ± 27.31 ^g,h^	5.63 ± 0.23 ^g^
E10	352.20 ± 22.01 ^e,f^	4.24 ± 0.01 ^e^
E11	454.54 ± 18.48 ^h^	3.35 ± 1.47 ^b,c,d,e^
E12	367.00 ± 9.45 ^f^	3.90 ± 1.25 ^d,e^
E13	333.80 ± 4.49 ^d,e^	2.54 ± 0.17 ^a,b,c^
E14	240.93 ± 10.31 ^a^	2.53 ± 0.25 ^a,b,c^
E15	287.41 ± 7.40 ^c^	2.53 ± 0.21 ^a,b,c^

Mean values within a column with the same letter are not significantly different at *p* = 0.05 using Duncan’s test.

**Table 3:** Process efficiency.

N1	N2	N3	N4	N5	N6	N7	N8	N9
0.39%	55.59%	48.37%	48.79%	45.68%	34.12%	50.69%	23.84%	71.29%

**Table 4:** Systems diameter.

	Diameter [nm]
N1	2470.56 ± 4.85 ^i^
N2	293.51 ± 0.84 ^a^
N3	2085.28 ± 3.46 ^h^
N4	309.09 ± 1.58 ^b^
N5	962.99 ± 0.60 ^e^
N6	481.17 ± 1.34 ^d^
N7	1755.84 ± 8.16 ^f^
N8	2031.17 ± 1.31 ^g^
N9	347.62 ± 0.78 ^c^

Mean values within a column with the same letter are not significantly different at *p* = 0.05 using Duncan’s test.

**Table 5:** Salidroside and rosarin content in the systems.

No.	Salidroside Content [μg/1 mg of the System]	Rosarin Content [μg/1 mg of the System]
N1	2.95 ± 0.03 ^d^	4.88 ± 0.25 ^e^
N2	2.23 ± 0.20 ^e^	3.52 ± 0.28 ^e^
N3	2.93 ± 0.05 ^d^	4.30 ± 0.27 ^a^
N4	3.02 ± 0.23 ^d^	4.47 ± 0.32 ^a,b^
N5	3.27 ± 0.25 ^c,d^	4.79 ± 0.38 ^a,b,c^
N6	3.69 ± 0.03 ^b^	5.41 ± 0.03 ^b,c,d^
N7	4.52 ± 0.42 ^a^	6.74 ± 0.38 ^c,d^
N8	3.44 ± 0.31 ^b,c^	5.23 ± 0.46 ^d^
N9	2.14 ± 0.10 ^e^	3.13 ± 0.18 ^f^

Mean values within a column with the same letter are not significantly different at *p* = 0.05 using Duncan’s test.

**Table 6:** Parameters of mathematical models fitted to the salidroside release profiles from systems 1–9.

	Mathematical Model									
No.	Zero-Order Kinetic		First-Order Kinetic		Higuchi Kinetic		Korsmeyer-Peppas Kinetic		Weibull Model	
	K	R^2^	K	R^2^	K	R^2^	R^2^	*n*	α	β
1	-	-	-	-	-	-	-	-	-	-
2	49.97	0.39	2.52	0.31	**14.46**	**0.71**	0.64	0.78	1.92	0.14
3	41.00	0.41	2.41	0.32	**11.71**	**0.72**	0.65	0.75	40.50	0.11
4	46.20	0.39	2.48	0.32	**13.37**	**0.71**	0.65	0.77	5.70	0.13
5	42.06	0.38	2.42	0.31	**12.36**	**0.69**	0.64	0.76	11.71	0.09
6	30.19	0.28	2.23	0.29	**9.69**	**0.62**	**0.62**	**0.71**	3.10	0.60
7	26.54	0.78	**3.55**	**0.81**	3.99	0.61	0.64	0.54	81.61	3.51
8	85.79	0.91	**5.25**	**0.85**	**14.07**	**0.79**	0.78	0.89	45.99	1.98
9	35.09	0.26	2.31	0.28	**11.55**	**0.60**	**0.62**	**0.74**	37.73	2.35

The best fit to the model is bolded; ‘-‘ – no data

**Table 7:** Permeability coefficient of salidroside and rosarin from systems and extract.

No.	Permeability Coefficient of Salidroside P_app_ × 10^−6^ [cm/s]	Permeability Coefficient of Rosarin P_app_ × 10^−6^ [cm/s]
N1	1.34 ± 0.05 ^c^	1.01 ± 0.02 ^c^
N2	1.94 ± 0.09 ^e^	1.06 ± 0.03 ^c,d^
N3	2.60 ± 0.08 ^g^	1.17 ± 0.02 ^d^
N4	1.65 ± 0.10 ^d^	2.09 ± 0.07 ^a,b^
N5	2.34 ± 0.07 ^f^	2.15 ± 0.07 ^a,b^
N6	2.91 ± 0.13 ^a^	2.20 ± 0.12 ^b^
N7	2.87 ± 0.09 ^a^	2.07 ± 0.02 ^a^
N8	3.22 ± 0.29 ^h^	2.09 ± 0.05 ^a,b^
N9	3.56 ± 0.06 ^i^	2.17 ± 0.12 ^a,b^
Extract	0.02 ± 0.01 ^b^	0.16 ± 0.02 ^e^

Mean values within a column with the same letter are not significantly different at *p* = 0.05 using Duncan’s test.

**Table 8:** Mucoadhesive properties.

No.	Component of Bioadhesion [cps]
N1	3.00 ± 0 ^c^
N2	3.50 ± 0 ^b^
N3	4.0 ± 0 ^a^
N4	2.17 ± 0.29 ^d^
N5	3.00 ± 0 ^c^
N6	2.33 ± 0.29 ^d^
N7	1.33 ± 0 ^f^
N8	1.50 ± 0 ^f^
N9	1.83 ± 0.29 ^e^

Mean values within a column with the same letter are not significantly different at *p* = 0.05 using Duncan’s test.

**Table 9:** Antioxidant and anti-inflammatory activity of system.

No.	Antioxidant Activity IC_50_ [mg/mL]	Anti-inflammatory Activity IC_50_ [mg/mL]
N1	25.76 ± 1.63 ^f^	3.2 ± 0.1 ^b^
N2	11.80 ± 0.61 ^b^	70.64 ± 5.07 ^e^
N3	8.54 ± 0.49 ^a^	26.52 ± 1.68 ^d^
N4	22.77 ± 1.30 ^e^	12.86 ± 1.24 ^a^
N5	11.56 ± 0.71 ^b^	12.54 ± 0.77 ^a^
N6	8.02 ± 0.52 ^a^	24.20 ± 1.42 ^c,d^
N7	35.65 ± 1.42 ^g^	22.67 ± 1.86 ^c^
N8	14.14 ± 0.88 ^d^	15.04 ± 1.42 ^a^
N9	1.85 ± 0.11 ^c^	0.33 ± 0.01 ^b^

Mean values within a column with the same letter are not significantly different at *p* = 0.05 using Duncan’s test.

**Table 10:** Factorial extraction process experiment plan.

No.	% of Methanol in the Extraction Mixture	Temperature [°C]	Time [min]
E1	50	30	60
E2	80	30	60
E3	50	70	60
E4	80	70	60
E5	50	50	30
E6	80	50	30
E7	50	50	90
E8	80	50	90
E9	65	30	30
E10	65	70	30
E11	65	30	90
E12	65	70	90
E13	65	50	60
E14	65	50	60
E15	65	50	60

**Table 11:** Factorial nanofibers production process experiment plan.

No.	CD Type *	CD Amount [g]	PVP Amount [g]
F1	1	1	1
F2	1	2	3
F3	1	3	2
F4	2	1	3
F5	2	2	2
F6	2	3	1
F7	3	1	2
F8	3	2	1
F9	3	3	3

* where (1) Hydroxyprodyl-α-cyclodextrin (HPαCD); (2) Hydroxyprodyl-β-cyclodextrin (HPβCD); (3) Hydroxyprodyl-γ-cyclodextrin (HPγCD)

## Data Availability

Data are contained within the presented article.
